# Evaluation of the effect of d-amino acid incorporation into amyloid-reactive peptides

**DOI:** 10.1186/s12967-017-1351-0

**Published:** 2017-12-11

**Authors:** Emily B. Martin, Angela Williams, Tina Richey, Craig Wooliver, Alan Stuckey, James S. Foster, Stephen J. Kennel, Jonathan S. Wall

**Affiliations:** 1 Department of Medicine, University of Tennessee Medical Center, 1924 Alcoa Hwy, Knoxville, TN 37920 USA; 2 Department of Radiology, University of Tennessee Medical Center, 1924 Alcoa Hwy, Knoxville, TN 37920 USA

**Keywords:** Amyloidosis, Peptides, p5, d-Amino acids, Imaging, Dehalogenation

## Abstract

**Background:**

Systemic amyloidoses comprise diseases characterized by the deposition of proteinaceous material known as amyloid. Currently, without performing multiple biopsies, there is no way to ascertain the extent of amyloid deposition in patients—a critical piece of information that informs prognosis and therapeutic strategies. We have developed pan-amyloid-targeting peptides for imaging amyloid and recently have adapted these for use as pre-targeting agents in conjunction with immunotherapy. Incorporation of d-amino acids in these peptides may enhance serum half-life, which is an important characteristic of effective peptide therapeutics. Herein, we assess the effects of partial incorporation of d-amino acids into the amyloidophilic peptide p5 on in vivo amyloid reactivity.

**Methods:**

Peptides, referred to as ^AQA^p5_(d)_, ^aqa^p5, and ^AQA^p5, were radiolabeled with iodine-125 and the tissue biodistribution (% injected dose/gram) measured in healthy mice at multiple time points post-injection. Microscopic distribution of the peptides was further visualized using microautoradiography (ARG). Peptides ^aqa^p5 and ^AQA^p5 were injected into healthy and amyloid-laden mice and evaluated by using SPECT/CT imaging at 1, 4 and 24 h post injection.

**Results:**

Biodistribution data and ARG revealed persistent retention of [^125^I]^AQA^p5_(d)_ in the liver and kidneys of healthy mice for at least 24 h. In contrast, peptides [^125^I]^aqa^p5 and [^125^I]^AQA^p5 did not bind these organs and was significantly lower than [^125^I]^AQA^p5_(d)_ at 24 h post injection (p < 0.0001). SPECT/CT imaging of amyloid-laden mice revealed accumulation of both [^125^I]^aqa^p5 and [^125^I]^AQA^p5 in amyloid-affected organs; whereas, in healthy mice, [^125^I]^aqa^p5 was observed in the kidneys and liver at early time points, and free radioiodide liberated during catabolism of [^125^I]^AQA^p5 was seen in the stomach and thyroid. Autoradiography confirmed that both [^125^I]^aqa^p5 and [^125^I]^AQA^p5 peptides specifically bound amyloid with no off-target binding to healthy organs.

**Conclusion:**

Incorporation of d-amino acids in amyloid-binding regions of amyloidophilic peptides resulted in off-target binding; however, N-terminus placement retained amyloid-specificity and evasion of deiodinases. Peptide ^aqa^p5, or similar reagents, may prove useful in novel immunotherapy strategies as well as for imaging renal, gastric and pancreatic amyloidosis.

**Electronic supplementary material:**

The online version of this article (10.1186/s12967-017-1351-0) contains supplementary material, which is available to authorized users.

## Background

The systemic amyloidoses are a group of diseases that result in the accumulation of misfolded proteins as fibrils in various organs and tissues. To date, more than 35 different proteins have been identified as fibrillar components of amyloid deposits in man [1–3]. In addition to protein fibrils, amyloid deposits also invariably contain hypersulfated heparan sulfate proteoglycans (HSPG) and serum proteins, notably serum amyloid P component, which are pathognomonic features of these diseases [3, 4]. In systemic forms of amyloidosis, any or all abdominothoracic organs or tissues can be involved; however, the liver, spleen, nerves, intestines, kidneys and heart are most common, and the relentless accumulation of tissue amyloid contributes to morbidity and mortality [5].

Amyloid patients often present with diverse symptomology and varied patterns of organ involvement; thus, accurate and rapid diagnosis is difficult, and the prognosis and treatment options depend on the affected organs and the extent of amyloid load [5, 6]. Given the clinical heterogeneity of these diseases, it is important to discern the amyloid burden and organ involvement in each patient such that appropriate therapies can be administered. Currently, there are no methods, in the US, to quantitatively assess whole-body amyloid load in patients with the many diverse types of systemic amyloidosis other than obtaining biopsies from every tissue—an invasive, potentially dangerous, impractical approach that is prone to sampling error. Of the clinically available amyloid-targeting imaging agents, none is capable of detecting all types of amyloid in all organs. For example, the use of technetium-99 m-labeled bone-seeking agents is limited only to cardiac transthyretin-associated amyloidosis [7]. Thus, there is an unmet clinical need for an imaging agent capable of detecting many, if not all, types of systemic amyloid regardless of the anatomic site of deposition. For this reason, our recent efforts have focused on developing an imaging agent for the non-invasive, quantitative assessment of amyloid load in patients [8–10].

To this end, we have developed synthetic, polybasic, amyloid-reactive peptides to serve as pan-amyloid imaging agents [8–12]. Since hypersulfated HSPG and protein fibrils—both linear arrays of charged components—are ubiquitously present in all amyloid fibrils, we designed polybasic peptides, which have been shown to bind specifically to amyloid via electrostatic components. One of the most highly characterized of these peptides, known as p5 [8, 12], is composed of 31 amino acids containing 4 amyloid-binding heptad repeats and a short leader sequence. Peptide p5, and related peptides, bind diverse forms of human amyloid extract and amyloid-like fibrils in vitro [11], and when radiolabeled via iodotyrosine, they have been shown to specifically bind systemic serum amyloid protein A-associated (AA) amyloid deposits in mice [8–12]. Recently, we have expanded the utility of the peptides as the basis of novel immunotherapy strategies, and peptides with an extended serum and target-bound half-life are desirable properties in our therapeutic agents. Consequently, we have explored different peptide structures containing d-amino acids that may confer increased serum half-life and provide resistance to dehalogenation of the iodotyrosine. The analog of p5 containing all d-amino acids, p5_(d)_, adopts an α-helical secondary structure and exhibited amyloid binding properties in vitro comparable to p5. However, when p5_(d)_ was radiolabeled and injected into amyloid-free mice, the peptide exhibited unexpected off-target binding, notably in the kidney and liver [10].

To further probe the effects of incorporating d-amino acids in amyloid-reactive peptides, we now describe studies of three variants that have been modeled after the 31-amino acid parent peptide, p5. The peptides evaluated in this study, designated ^AQA^p5_(d)_, ^aqa^p5 and ^AQA^p5, contain modified N-terminal amino acids, relative to the p5 peptide, to allow incorporation and evaluation of d-amino acids. These peptides, when radioiodinated, exhibited distinct biodistribution and dehalogenation propensity in healthy mice. The ^AQA^p5_(d)_ variant, with d-amino acids in the amyloid-reactive site, bound intensely to tubules in the renal cortex and throughout the liver of amyloid-free mice; whereas, peptide ^aqa^p5, with the incorporation of only three d-amino acids at the N-terminus, retained specific amyloid reactivity in vivo and prevented dehalogenation of the juxtaposed l-iodotyrosine. This allowed precise and accurate determination of the peptide’s tissue biodistribution without the complication of the loss of iodine-125 from the radiotracer. This peptide, ^aqa^p5, was shown to specifically bind AA amyloid deposits in mice and did not exhibit off-target reactivity in the liver or kidney. The importance of d-amino acid placement in amyloid-reactive peptides that may have utility in novel therapeutic paradigms or as imaging agents is discussed.

## Methods

### Peptide preparation

Peptides ^AQA^p5_(d)_, ^aqa^p5 and ^AQA^p5 (Table 1) were purchased from Anaspec (Fremont, CA) as crude preparations and were purified by reverse-phase high performance liquid chromatography (RP-HPLC; Biologic DuoFlow; BioRad, Hercules, CA) using a C3 matrix (ZorbaxTM 300SB; Agilent, Santa Clara, CA). A linear gradient of 1–51% acetonitrile in water with 0.05% v/v trifluoroacetic acid served as the mobile phase. A flow rate of 4 mL/min was used to elute the peptides. Fractions were collected, pooled, and integrity of the peptides was verified by mass spectrometry [9, 10]. Analytical HPLC was performed, essentially as described above, but using a Zorbax SB-C3 1500 mm matrix with a flow rate of 1 mL/min.

**Table 1:** Primary structure of peptides

Peptide	Primary structure
^AQA^p5_(d)_	AQAys kaqka qakqa kqaqk aqkaq akqak q
^aqa^p5	aqaYS KAQKA QAKQA KQAQK AQKAQ AKQAK Q
^AQA^p5	AQAYS KAQKA QAKQA KQAQK AQKAQ AKQAK Q

d-Amino acids are shown in lower case

### Circular dichroism

CD spectra were obtained for all peptides (0.05 mg/mL in PBS) using a DSM 1000 CD instrument (Olis Inc., Bogart, Georgia) with a peptide sample volume of 2.9 mL and a 1 cm cuvette path length. Data were collected in triplicate over the 190–250 nm wavelength range with 1 nm increments. Increasing amounts of low molecular weight heparin (Enoxaparin sodium, Sanofi, Bridgewater, NJ) up to 1.5 mg was added to each peptide to induce secondary structure transitions. All data were corrected for background by subtraction of a PBS or PBS with enoxaparin CD spectrum. Mean residue ellipticity [*Θ*] was calculated according to: *Θ* *(MW/no. AA)/(10 *conc. *l) where: *Θ* is ellipticity (millidegrees); MW is the molecular weight of the peptide (3356.8 Da); no. AA, is the number of amino acid residues, (31); conc. is the peptide concentration (mg/mL), and l, is the cuvette path length (cm).

### Radiolabeling

Peptides (20–40 μg) were radiolabeled with iodine-125 (^125^I; Perkin Elmer, Waltham, MA) as previously described [9]. Briefly, ~ 2 mCi of ^125^I in buffered sodium phosphate pH 7.6 was mixed with peptide and 20 μg chloramine T in a volume of 10 µL water for 1 min. The reaction was quenched using 20 μg sodium metabisulphite in 10 µL water. Radiolabeled product was separated from free isotope by gel filtration chromatography using a Sephadex G-25 size-exclusion matrix (PD10; GE Healthcare) equilibrated with 0.1% gelatin/PBS. Fractions were collected manually and radioactivity in each measured by gamma counting after which the peak fractions were pooled. Radiochemical purity was assessed qualitatively by estimating the free radioiodide from phosphor images (Cyclone Storage Phosphor System, PerkinElmer, Shelton, CT) of SDS-PAGE gels.

### Animal model

A transgenic murine model of systemic AA amyloidosis was used for this study [13]. The H2-L^d^-huIL-6 Tg Balb/c transgenic mice (H2/IL-6 mice) constitutively express the human interleukin-6 (IL-6) transgene resulting in a chronic inflammatory state and hepatic production of serum amyloid protein A. Amyloidosis was induced in these mice by intravenous (IV) injection of 10 μg of purified, splenic AA amyloid (amyloid enhancing factor, [14]) suspended in 100 μL of sterile phosphate-buffered saline (PBS; pH 7.4). Biodistribution and imaging studies were performed at 4–6 wk post-induction of amyloid at which point a significant amount was present in liver, spleen, pancreas, kidney, heart, stomach and intestines. All animal manipulation and experimental procedures were performed under the auspices of a protocol approved by the University of Tennessee Institutional Animal Care and Use Committee. The University of Tennessee Medical Center Animal Facility is a component of the University of Tennessee AAALAC-I-approved animal program.

### SPECT/CT imaging

SPECT/CT imaging was performed as described previously [9]. Briefly, amyloid-free, wild type (WT) mice or amyloid-laden mice (AA) were injected with ~ 5 μg of [^125^I]^aqa^p5 or [^125^I]^AQA^p5 (~ 150 μCi) in the lateral tail vein. After 1, 4, or 24 h, the mice were euthanized by an isoflurane inhalation overdose. SPECT images were acquired using an Inveon trimodality imaging platform (Siemens Preclinical Solution, Knoxville, TN, [15]). Low energy (^125^I; 25–45 keV) gamma photons were detected at each of 60, 16-s projections with 90 mm of bed travel in 1.5 revolutions. A 0.5 mm-diameter, 5-pinhole (mouse whole body) collimator was used at 30 mm from the center of the field of view. The image data were reconstructed using a 3D ordered subset expectation maximization (OSEM) algorithm (16 iterations, 6 subsets) and displayed onto a 88 × 88 × 244 matrix with 0.5-mm isotropic voxels. Zoom and β values of 1 were used.

CT data were acquired using an X-ray voltage biased to 80 kVp with a 500 μA anode current with an aluminum filter with 0.5 mm thickness. A 240 ms exposure was used, and 360, 1° projections were collected. The data were reconstructed using an implementation of the Feldkamp filtered back-projection algorithm onto a 256 × 256 × 603 matrix with isotropic 211.5 μm voxels. The data were down-sampled by a bin of 4. SPECT and CT datasets were co-registered and visualized using the Inveon Research Workplace software package (Siemens Preclinical Solution, Knoxville, TN).

### Biodistribution

Following image acquisition, a necropsy was performed to collect samples from 10 tissues (muscle, liver, pancreas, spleen, left and right kidneys, stomach, upper and lower intestines and heart). A small piece of each tissue was weighed in tared vials and radioactivity quantified using an automated gamma counter (1480 Wizard 3 Gamma Counter, Perkin Elmer). The tissue radioactivity data were expressed as % injected dose per gram of tissue (%ID/g). An additional sample of each tissue was fixed in buffered formalin for microautoradiographic studies.

### Microautoradiography and Congo red tissue staining

Six-micrometre-thick tissue sections were cut from formalin-fixed, paraffin-embedded material for microautoradiography. Sections were placed on Plus microscope slides (Fisher Scientific), dipped in NTB-2 emulsion (Eastman Kodak) and stored in the dark for 4 days. Thereafter, the emulsion was developed and tissues counterstained with hematoxylin and eosin. For the detection of amyloid, tissue sections were stained with an alkaline Congo red solution (0.8% w/v Congo red, 0.2% w/v KOH, 80% ethanol) for 1 h at room temperature followed by counterstain with Mayer’s hematoxylin for 2 min. Digital microscopic images were acquired using a Leica DM500 light microscope fitted with cross-polarizing filters (for Congo red birefringence) fitted with a cooled CCD camera (SPOT RT-slider, Diagnostic Instruments, Sterling Heights, MI).

### Preparation of mouse tissue extracts

Kidney or liver tissue was harvested from WT mice at necropsy. Approximately 0.5 g of tissue was removed and a tenfold volume of PBS, pH 7.4, added. Serine protease inhibitors, leupeptin and phenylmethylsulfonyl fluoride (PMSF), were added, each at 100 μg/mL. The solution was mixed vigorously (3 × 10 s bursts) with a Polytron (Kinematica Inc., Bohemia, NY) and then centrifuged at 4000×*g* for 10 min. The supernatant containing the soluble protein fraction was collected and centrifuged twice more, as above. The supernatant from the final wash was collected and sodium azide and tween-20 were both added at 0.05%.

### Ex vivo dehalogenation assay

Aliquots of radioiodinated peptide were diluted 1/50 in PBS with 0.05% tween-20 (PBST). Ten microliter of this solution was added to 200 μL of WT mouse tissue extract supernatants (liver or kidney) or mouse serum each prepared at 50% v/v in PBS. Peptide in 200 µL of PBST served as a control. Samples were incubated for 1 h at 37 °C. Five microliter of each reaction mixture were added to 7.5 μL gel sample loading buffer (4×; Invitrogen, Carlsbad, CA). Samples were loaded onto 4–12% Bis–Tris SDS-PAGE gels (Invitrogen) and electrophoresis performed for 25 min with MES buffer at 200 V. Phospohorimaging was performed with a 2 h exposure to visualize the bands in the gel.

### Statistics

Analysis of biodistribution data in WT mice was performed using a 2-way ANOVA with a Tukey correction for multiple comparisons and a family-wise significance level of *p* = 0.05. Analyses were performed using the Prism software package (v. 6.07 or greater; GraphPad Software Inc., La Jolla, CA).

## Results

### Peptide characterization

Analytical RP-HPLC revealed that, following purification, each peptide migrated as a single peak, with a retention time of ~ 9 min, indicating a lack of significant impurities (Additional file 1: Figure S1). A trailing shoulder was evident in each chromatogram; however, this represented ~ 10% of the total area under the curve.

**Fig. 1:**
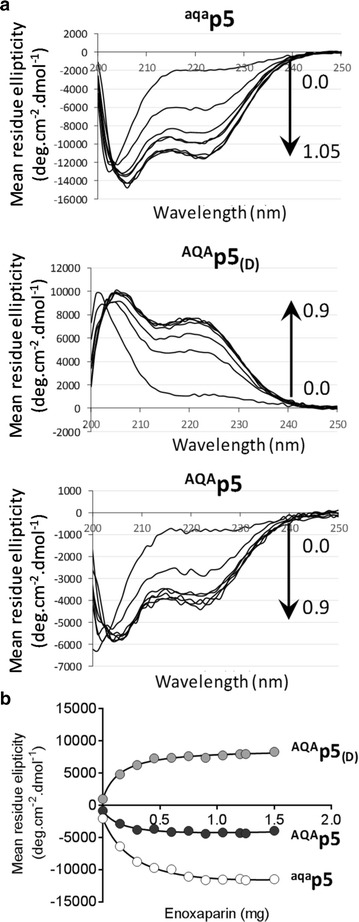
Peptides exhibit an α-helical secondary structure. **a** Circular dichroism spectra of peptides ^aqa^p5, ^AQA^p5_(d)_ and ^AQA^p5 adopt a helical secondary structure in the presence of low molecular weight heparin as evidenced by the change in mean residue ellipticity at 222 nm. **b** The heparin-mediated change in ellipticity at 222 nm yielded midpoints of 0.33, 0.15, and 0.19 mg for peptides ^aqa^p5, ^AQA^p5_(d)_ and ^AQA^p5, respectively

Circular dichroism revealed that the peptides, suspended in water at 0.05 mg/mL, adopted a predominantly coil structure with a minima/maxima at ~ 202 nm and no evident minima at 222 nm (Fig. 1a). Upon titration of low molecular weight heparin, up to 1.5 mg, minima/maxima at 222 nm were observed, indicative of the formation of helical structure (Fig. 1a). Evaluation of the transition in mean residue ellipticity at 222 nm yielded half maximal values of 0.33 mg, 0.15 mg and 0.19 mg for ^aqa^p5, ^AQA^p5_(d)_ and ^AQA^p5, respectively (Fig. 1b).

### In vivo distribution of peptides ^AQA^p5_(d)_, ^aqa^p5 and ^AQA^p5 in WT mice

**Fig. 2:**
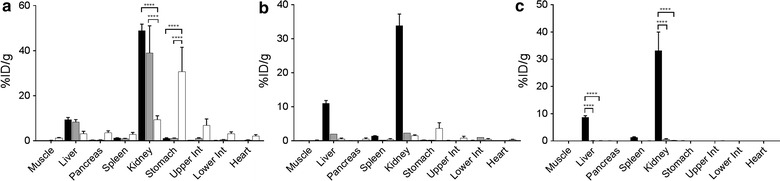
Biodistribution of radiolabeled peptide in healthy WT mice. The tissue distribution, expressed as percent inject dose per gram (%ID/g) of tissue, of radioiodinated peptides ^AQA^p5_(d)_, ^aqa^p5 and ^AQA^p5 was determined in WT mice by measuring tissue radioactivity at 1 h (**a**), 4 h (**b**) and 24 h (**c**) post injection. The data are expressed as mean ± SD (n = 3) and were analyzed using ANOVA with multiple comparisons (****p < 0.0001)

To examine the in vivo dehalogenation of the three peptides, they were radiolabeled and injected into healthy wild type (WT) mice that contain no target amyloid. The three peptides were readily radiolabeled and, after purification, were deemed to be > 90% pure based on analysis of the phosphorimaging (data not shown). The biodistribution of radioiodinated peptides ^AQA^p5_(d)_, ^aqa^p5 and ^AQA^p5 in vivo was initially examined in healthy, amyloid-free WT mice at 1, 4 and 24 h post injection (Fig. 2). At 1 h, tissue uptake was dominated by the liver, kidneys and stomach (Fig. 2a). Both [^125^I]^AQA^p5_(d)_ and [^125^I]^aqa^p5 had threefold greater retention in the liver, as compared to [^125^I]^AQA^p5; however, this difference did not achieve statistical significance. In the kidneys, we observed significantly greater amounts of radiolabeled ^AQA^p5_(d)_ (~ 45%ID/g) and ^aqa^p5 (~ 39%ID/g) as compared to [^125^I]^AQA^p5 (9%ID/g).

In contrast, there was significantly more radioiodide in the stomach of mice administered [^125^I]^AQA^p5 (30.7%ID/g) relative to [^125^I]^AQA^p5_(d)_ and [^125^I]^aqa^p5, where only ~ 1%ID/g was observed in each case (p < 0.0001). This is indicative of the accumulation of free radioiodide liberated during catabolism of peptide [^125^I]^AQA^p5 but not [^125^I]^AQA^p5_(d)_ or [^125^I]^aqa^p5. At 4 and 24 h (Fig. 2b, c), the levels of radioactivity in all organs of mice administered [^125^I]^aqa^p5 and [^125^I]^AQA^p5 had decreased. They decreased further at 24 h post injection, such that all values were < 0.5%ID/g (Fig. 1c). This differed dramatically from the mice receiving [^125^I]^AQA^p5_(d)_ in whom the liver (~ 9%ID/g) and kidneys (~ 40%ID/g) retained significantly higher levels of radioactivity (Fig. 2c).

**Fig. 3:**
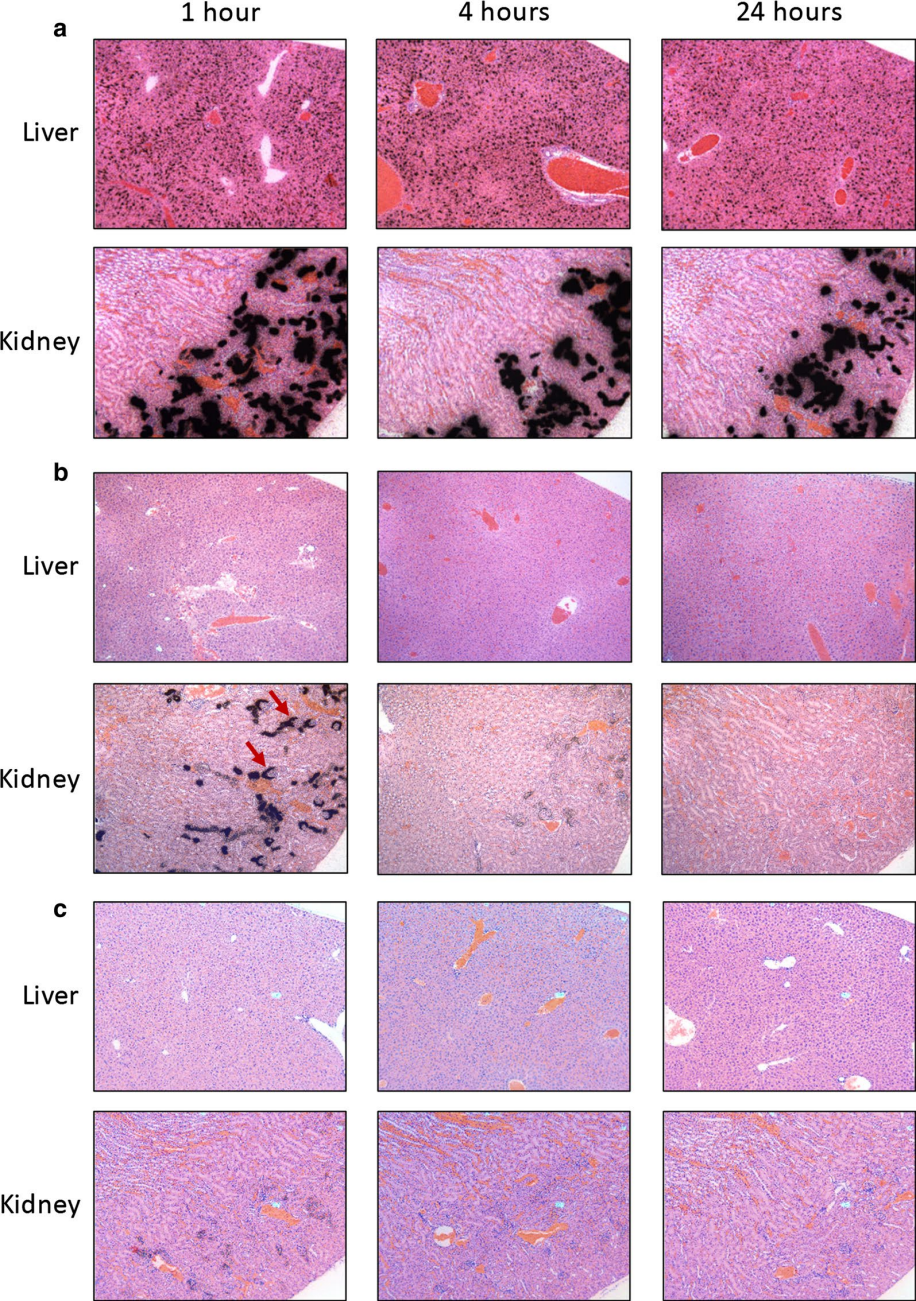
Microdistribution of radiolabeled peptides revealed liver and kidney retention of ^AQA^p5_(d)_ but not of the other two peptides. **a** Peptide ^AQA^p5_(d)_ was observed microautoradiographically, as evidenced by the presence of black silver grains, in the liver and renal cortex at 1, 4, and 24 h post injection. Peptides ^aqa^p5 (**b**) and ^AQA^p5 (**c**) were apparent in the renal cortex at 1 h post injection during catabolism, but their presence decreased at 4 and 24 h post injection. There was no evidence of retention of either peptide in the liver at any time point

Since biodistribution measurements revealed that the significant differences in radiotracer retention in WT mice were in the liver and kidneys, microautoradiography of these tissues was used to visualize the microdistribution of each of the peptides at 1, 4 and 24 h post injection. Radiolabeled peptide was evidenced in the microautoradiographs by the presence of black silver grains (Fig. 3). Consistent with our tissue biodistribution data of [^125^I]^AQA^p5_(d)_, radioactivity was observed as punctate deposits throughout the WT mouse liver and did not appear to diminish over the 24 h post injection (Fig. 3a). Similarly, in the kidney of these mice, dense, focal radioactivity deposits were observed exclusively within the renal cortex at 1 h post injection and persisted for 24 h (Fig. 3a). The distribution of [^125^I]^aqa^p5 in the liver was, in contrast, diffuse and barely visible microscopically when viewed at low magnifications, even at 1 h post injection (Fig. 3b). In the kidney, radioactivity was observed to be less dense, as compared to mice receiving [^125^I]^AQA^p5_(d)_, and distributed both in the renal cortex and medulla. The radioactivity was associated with Bowman’s space (arrows) as well as renal proximal tubules. The intensity of the signal decreased rapidly, and by 24 h post injection, only a diffuse, uniform distribution was visible throughout the kidney (Fig. 3b). The microdistribution of radioactivity in the mice that received [^125^I]^AQA^p5 (Fig. 3c) was similar to that for [^125^I]^aqa^p5; however, the presence of silver grains was greatly diminished in the liver and kidney, even at 1 h post injection (Fig. 3c).

In vitro *dehalogenation* To assess whether dehalogenation of peptides was associated with a soluble enzyme in the liver or kidney, each peptide was incubated in supernatants prepared from these organs, as well as serum, and the liberation of free radioiodide monitored by gel electrophoresis. After 1 h incubation, no free iodide was detected in any peptide sample under any conditions (data not shown). This indicates that in vivo dehalogenation of these peptides is a complex process, possibly involving membrane bound enzymes.

### SPECT/CT imaging of peptides ^aqa^p5 and ^AQA^p5 in AA and WT mice

**Fig. 4:**
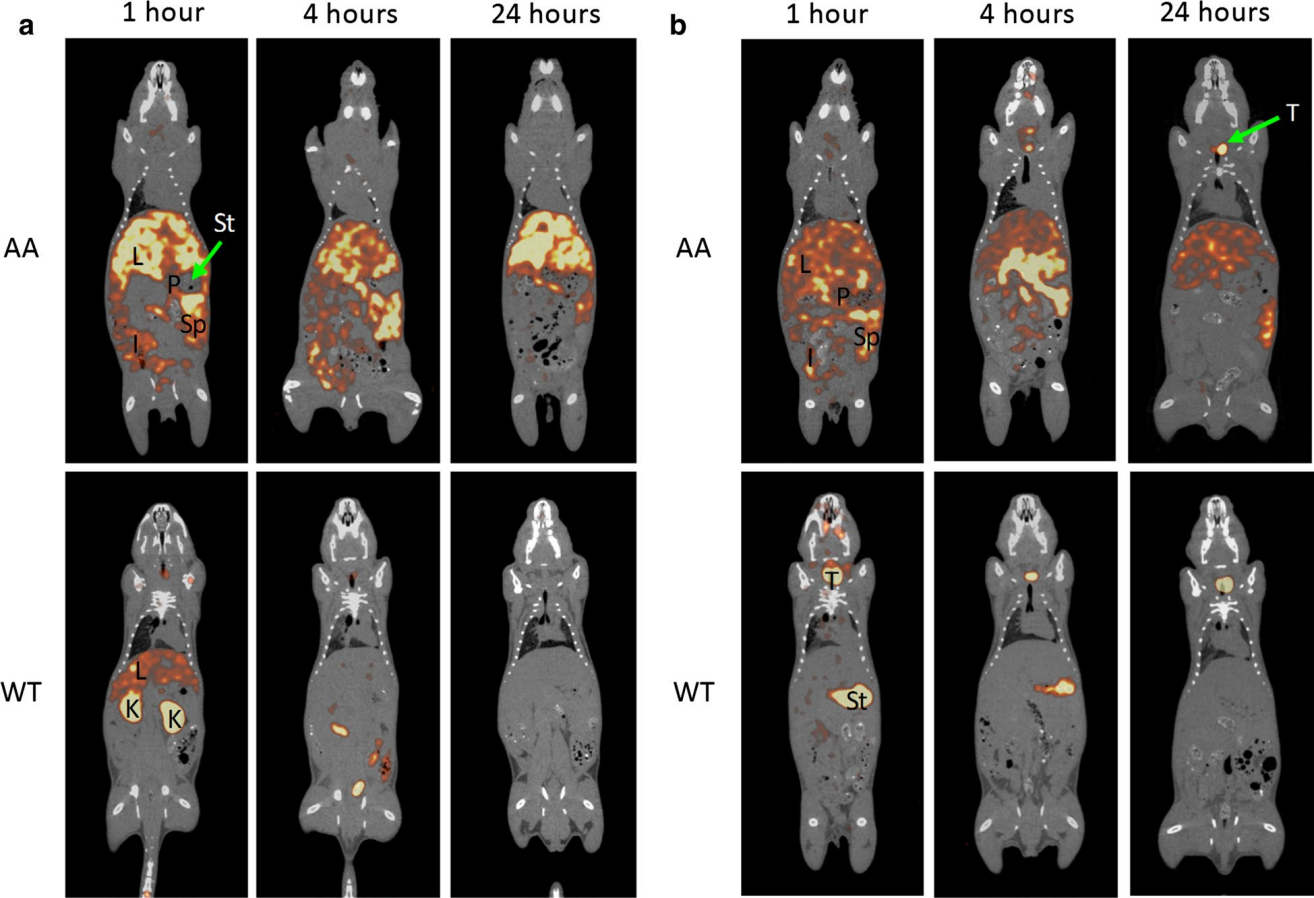
SPECT/CT imaging of radioiodinated ^aqa^p5 and ^AQA^p5 in AA and healthy mice. **a** Radiolabeled ^aqa^p5 was observed in the liver (L), spleen (Sp), pancreas (P) and intestine of mice with AA amyloid which persisted for more than 24 h post injection. In contrast, in WT mice, [^125^I]^aqa^p5 was observed in the hepatic blood pool and kidney (K) at 1 h but was excreted and not visible at 4 h post injection. **b** [^125^I]^AQA^p5 was observed in the liver, spleen, pancreas and intestine of AA mice; whereas, in WT mice free radioiodide liberated during catabolism was observed in the stomach (St) and thyroid (T)

Radioiodinated peptide ^AQA^p5_(d)_ exhibited significant binding to healthy, amyloid-free tissues in WT mice and is, thus, not useful as an agent for specifically targeting amyloid. The remaining two peptides, [^125^I]^aqa^p5 and [^125^I]^AQA^p5, were evaluated for specific amyloid reactivity in mice with AA-associated amyloidosis (Fig. 4). Small animal SPECT/CT imaging of [^125^I]^aqa^p5 and [^125^I]^AQA^p5 in AA mice at 1, 4, and 24 h revealed accumulation of the radiotracter in abdominal organs associated with amyloid deposition in this mouse model—notably, the liver (L), spleen (Sp), pancreas (P), and intestines (I). There was no evidence of radioactivity in the stomach (St) of mice administered [^125^I]^aqa^p5 (Fig. 4a), supporting the hypothesis that [^125^I]^aqa^p5 is not significantly dehalogenated. Qualitative comparison of the signal intensity of the SPECT images of [^125^I]^aqa^p5 and [^125^I]^AQA^p5 (Fig. 4b) suggests that ^aqa^p5 was retained, particularly by the liver, in greater amounts and persisted longer than ^AQA^p5; however, the amount of amyloid in each of these mice may have been different. In the mice receiving [^125^I]^AQA^p5, radioactivity was detected in the thyroid (T) at 4 and 24 h post injection (Fig. 4b)—this was less evident in mice administered [^125^I]^aqa^p5 (Fig. 4a).

Imaging of [^125^I]^aqa^p5 in WT mice showed diffuse and concentrated accumulation of the radiotracer in the liver (L) and kidney (K), respectively, at 1 h post injection. At 4 and 24 h post injection, only sparse intestinal radioactivity was observed in these mice (Fig. 4a). In contrast, the stomach (St) and thyroid (T) were the major sites of radioactivity at 1 h post injection in mice receiving [^125^I]^AQA^p5 (Fig. 4b). In these mice, the stomach activity was lost over 24 h, whereas the thyroid radioactivity persisted. At 24 h post injection, with the exception of the thyroid, there was no evidence of binding, by SPECT imaging, of [^125^I]^aqa^p5 or [^125^I]^AQA^p5 to healthy organs or tissues (Fig. 4).

### Microautoradiography of [^125^I]^aqa^p5 and [^125^I]^AQA^p5 in AA-laden tissue

**Fig. 5:**
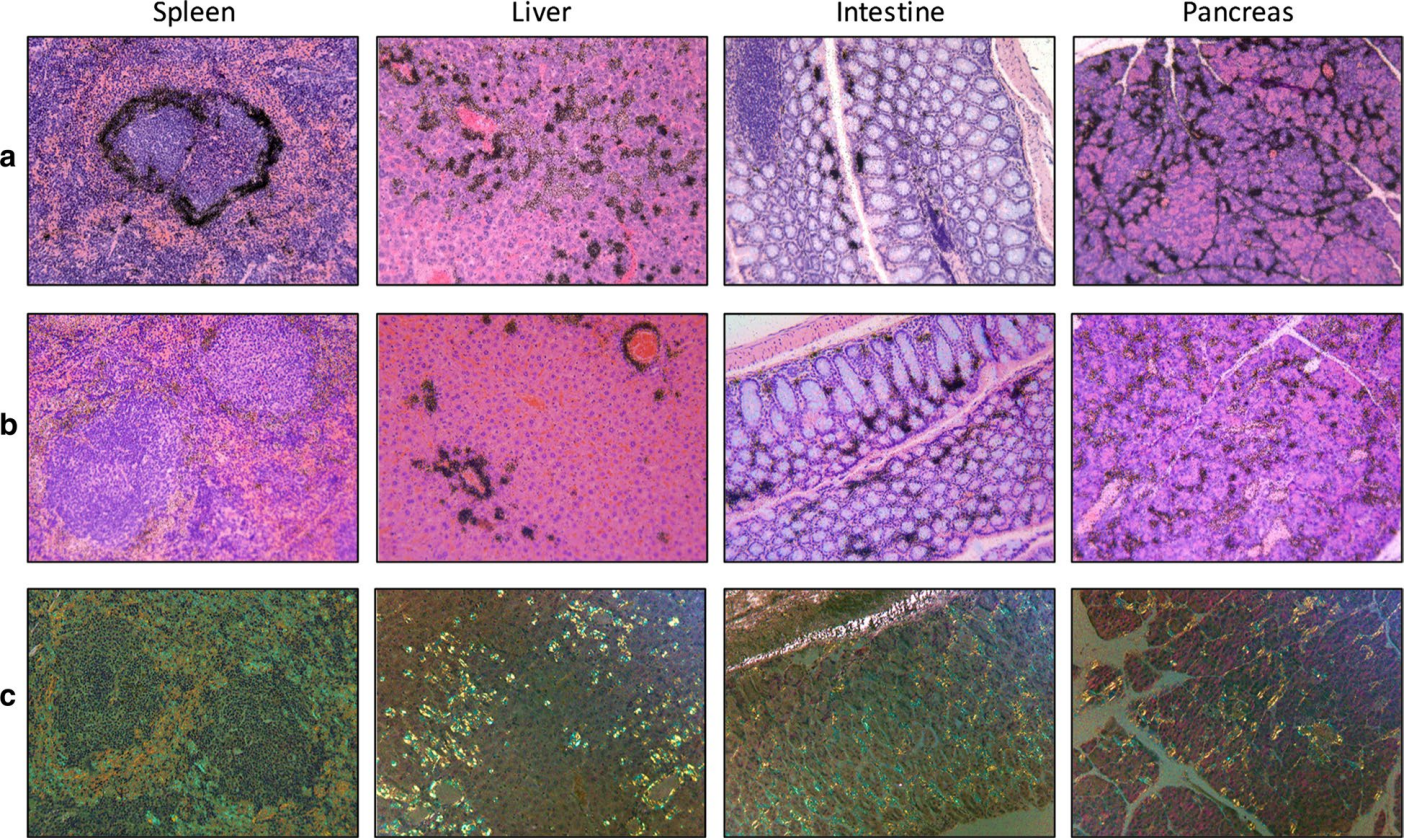
Specific amyloid binding of radiolabeled peptides ^aqa^p5 and ^AQA^p5 in AA mice. Both [^125^I]^aqa^p5 (**a**) and [^125^I]^AQA^p5 (**b**) localized with AA amyloid in the spleen, liver, intestine, and pancreas as evidenced microautoradiographically, by the presence of black silver grains at sites of amyloid deposition. **c** The characteristic distribution of amyloid in the organs was evidenced by the presence of green–gold birefringent amyloid in Congo red-stained tissue sections

To confirm specific binding of peptides [^125^I]^aqa^p5 and [^125^I]^AQA^p5 to amyloid deposits in the organs, microautoradiographic analysis of the spleen, liver, intestine and pancreas was performed. The distribution of black silver grains, representing the presence of [^125^I]^aqa^p5 (Fig. 5a) and [^125^I]^AQA^p5 (Fig. 5b), was compared to the amyloid distribution, evidenced by the green-gold birefringence in Congo red-stained tissue sections (Fig. 5c). Representative tissue sections are shown to provide patterns of amyloid distribution in these organs (Fig. 5c). The microdistribution of [^125^I]^aqa^p5 in the spleen, liver, intestine, and pancreas (Fig. 5a) was discrete and consistent with the pattern of amyloid distribution. Similarly, the distribution of [^125^I]^AQA^p5 was consistent with amyloid binding (Fig. 5b), although to a lesser extent as compared to [^125^I]^aqa^p5 given that the silver deposits were less dense, notably in the spleen and pancreas.

## Discussion

To date, our studies of amyloid-reactive peptides in vivo have focused almost exclusively on variants that are radioiodinated via the l-tyrosine residue at position 4. These peptides are susceptible to catabolic dehalogenation in the liver and kidney [16, 17] that can complicate interpretation of the peptide biodistribution data. However, during these studies, we have recognized that dehalogenation of unbound peptide can be exploited in medical imaging for specific detection of renal and hepatic amyloidosis when image data are collected at later time points post injection [8]. Nevertheless, we now seek to exploit these peptides for treatment of amyloidosis, and incorporation of d-amino acids for the enhancement of in vivo stability and lifetime in the circulation may be useful if amyloid-specific reactivity can be maintained.

Incorporation of non-natural, d-amino acids into synthetic peptides has been shown to render them more resistant to proteolysis and vascular clearance in vivo as compared to their l-amino acid counterparts [18–20]. Increased bioavailability, a valuable asset for peptide therapeutics, is one advantage of extending the vascular half-life of peptides. The peptidomimetic bradykinin antagonist *Icatibant*™ incorporates d-amino acids to inhibit proteolytic digestion, and it is approved for the treatment of hereditary angioedema [21]. Additionally, a retro-inverso peptide inhibitor of Aβ aggregation exhibits a prolonged half-life and enhanced inhibitory efficacy as compared to the l-amino acid homolog [19, 22]. However, placement of d-amino acids in a bioactive peptide is critical to ensure that binding specificity and bioactivity is not jeopardized. Inappropriate substitution of d-amino acids in the center of an antimicrobial peptide sequence resulted in a complete loss of activity [23]. Nonetheless, incorporation of d-amino acids that flank the “active” site can be advantageous [24].

Our previous evaluation of the all d-amino acid peptide, p5_(d)_, in amyloid-free mice demonstrated that, despite retaining excellent amyloid-reactivity in in vitro assays, the peptide bound to amyloid-free tubules in the renal cortex (but not the glomerulae) and a diffuse ligand throughout the liver [10]. Binding at these sites persisted for at least 24 h post injection, indicating that the all d-enantiomer variant of the amyloidophilic peptide p5 was unsuitable for specific amyloid-targeting in vivo.

To further probe the effect of d-amino acids in homologs of peptide p5, a novel tripeptide Ala-Gln-Ala was substituted for Gly-Gly-Gly- to facilitate assessment of d-amino acids at the N-terminal, which has been reported to be beneficial [25]. Since there is no d-enantiomer of glycine, Ala-Gln-Ala, which constitutes the spacing motif within the heptad repeat of peptide p5, was used at the N-terminal and, thus, maintained consistency of the amino acid sequence. Incorporation of d-amino acids at the N-terminal has been shown to enhance electrostatic stability of helical peptides [26] and increase the helicity of peptides composed of amino acids with an intrinsic propensity for helical structure in aqueous milieu [27]. The increased propensity to form an alpha helix in amyloid-reactive, polybasic peptides has been shown to enhance the affinity for amyloid deposits in vivo [12].

Using an N-terminal Ala-Gln-Ala composed of d-amino acids, peptide ^aqa^p5 was mostly resistant to dehalogenation, as evidenced by the lack of radioiodide in the stomach, even though the Tyr residue remained an l-enantiomer (Fig. 2). Thus, the presence of d-amino acids on the N-terminal side of the l-iodotyrosine largely prevented dehalogenation of the peptide by deiodinases in the liver and kidney. Based on these data, we speculate that murine deiodinases require l-amino acids adjacent to the iodotyrosine during enzymatic dehalogenation for activity. Thus, juxtaposed d-enantiomers may sterically hinder the interaction between the deiodinase active site and the l-iodotyrosine. Since this peptide is mostly resistant to dehalogenation, it can be effectively tracked in vivo. These data provide the first indication that this class of polybasic α-helical peptides, which includes p5 [8, 12] and p5 + 14 [9], does not bind intracellular ligands along the catabolic pathway within the renal proximal tubule or hepatobiliary system. Furthermore, [^125^I]^aqa^p5 retained specific amyloid-binding in mice with severe systemic AA-associated amyloidosis, such that amyloid in the liver, spleen, pancreas and intestine was readily detected by SPECT imaging and microautoradiography at least 24 h post injection. Despite the previously reported beneficial effect of peptide dehalogenation in amyloid imaging by peptides p5 and p5 + 14 [8, 9], in the case of peptide ^aqa^p5, resistance to dehalogenation could be advantageous particularly for the detection of gastric or pancreatic amyloid due to the absence of free radioiodide sequestered by the stomach. Therefore, radioiodinated peptide ^aqa^p5, or a similar reagent, could provide a novel tool for imaging amyloid deposits in the kidney, pancreas and stomach.

Comparison of biodistribution of the halogen-retaining peptides ^AQA^p5_(d)_ and ^aqa^p5 in healthy WT mice demonstrated gross differences. Peptide [^125^I]^AQA^p5_(d)_ was resistant to dehalogenation due to the incorporation of a d-Tyr at position four. This peptide was sequestered rapidly by kidney and liver and was retained there for at least 24 h (Figs. 1, 2). This is consistent with the biodistribution of the all d-amino acid peptide, p5_(d)_, and suggests that d-amino acid incorporation in the amyloid-binding heptad repeat region is responsible for the off-target reactivity in vivo. Although the precise nature of the ligand recognized by both ^AQA^p5_(d)_ and p5_(d)_ in healthy, amyloid-free tissue remains uncharacterized, it is possible that the ligand is a sulfated, membrane surface-bound proteoglycan, such as heparan sulfate proteoglycan. The distribution is reminiscent of [^125^I]-labeled basic fibroblast growth factor (bFGF), particularly within the sinusoids of the liver [28]. Although, in the kidneys, [^125^I]bFGF bound avidly to glomerular tufts rather than the proximal tubules as seen in mice administered [^125^I]^AQA^p5_(d)_. In contrast, [^125^I]^aqa^p5 was not retained in the liver or kidney at 4 and 24 h post-injection.

The all l-amino acid peptide, ^AQA^p5, also exhibited specific amyloid-binding capabilities, albeit with qualitatively less radiotracer uptake in the liver and spleen as compared to [^125^I]^aqa^p5. However, this cannot be attributed with certainty to a single phenomenon since this peptide is prone to dehalogenation, and the AA mice used in this study may have had differences in amyloid load.

## Conclusions

We have studied the effects of d-amino acid placement in amyloid-reactive peptides in anticipation that this strategy might be useful to enhance the proteolytic stability and enhance the biological half-life of peptide variants for use in novel immunotherapy strategies. Our findings indicate that: (1) complete incorporation of d-amino acids in the amyloid-binding heptad repeat caused loss of amyloid-binding specificity in vivo due to competitive retention in the liver and kidneys—sites of peptide catabolism. (2) In contrast, placement of d-enantiomers at the peptide N-terminus did not inhibit amyloid-binding specificity. (3) This placement of d-amino acids provided resistance against the activity of deiodinases. Thus, appropriate placement of d-amino acids, e.g., at the N-terminus, could provide a reagent (^aqa^p5) with enhanced in vivo stability and resistance to dehalogenation. Such a reagent may serve as not only a potential candidate for amyloid immunotherapy strategies but also as a novel candidate for amyloid imaging with specific advantages for the detection of gastric, renal, or pancreatic amyloid.

## Additional file

**Additional file 1: Figure S1.** Purified peptides appeared, by RP-HPLC chromatography, as a single peak with a retention time of ~ 9 min

## Abbreviations

AA: serum amyloid protein A-associated amyloidosis
[^125^I]: iodine-125
SPECT/CT: single photon emission computed tomography/X-ray computed tomography
ARG: microautoradiography
