# Supplementary figures and images for: Evaluation of the effect of d-amino acid incorporation into amyloid-reactive peptides
Source: J Transl Med. 2017 Dec 11;15:247. doi: 10.1186/s12967-017-1351-0 (PMC5725832; doi:10.1186/s12967-017-1351-0)

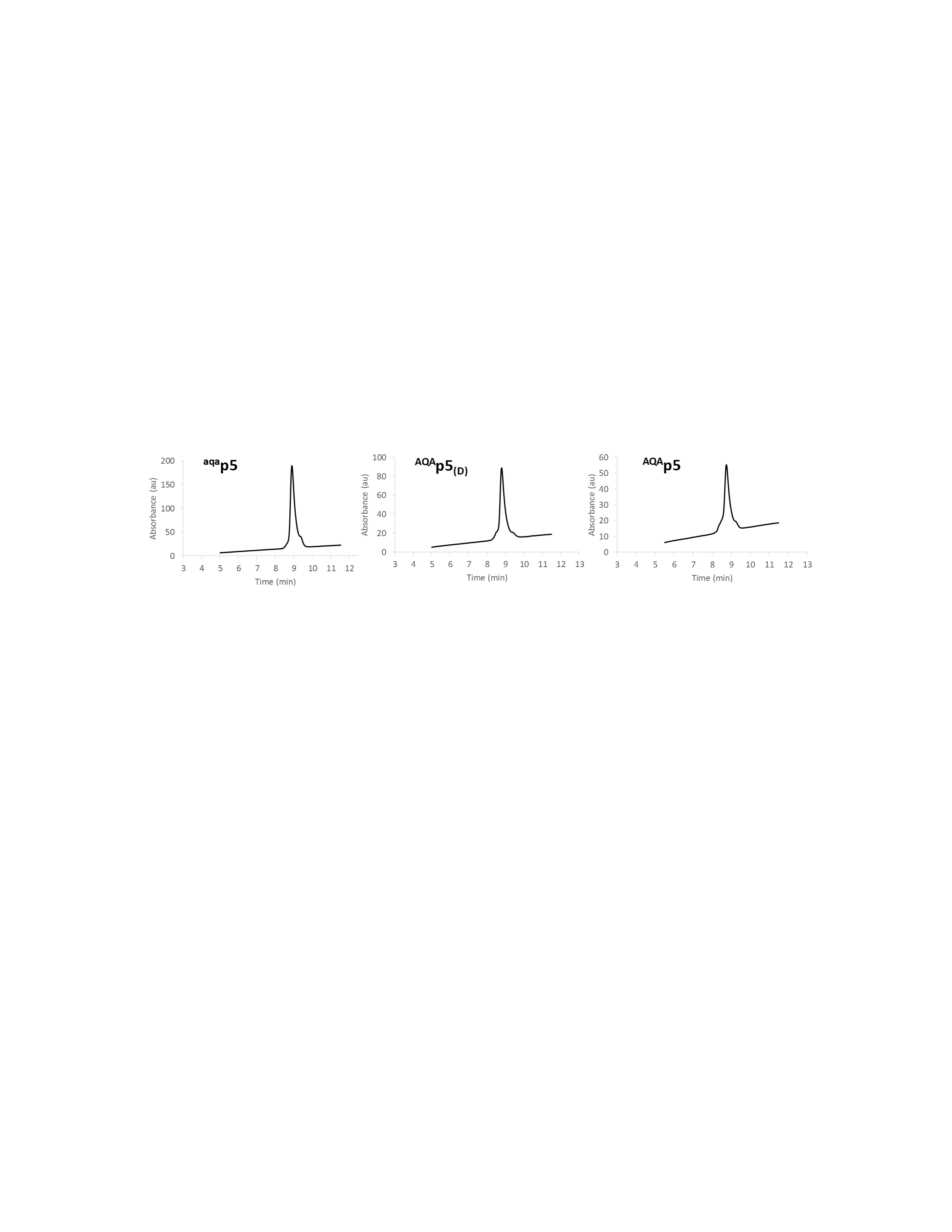

Supplement: Supplementary file 1 — Additional file 1: Figure S1. Purified peptides appeared, by RP-HPLC chromatography, as a single peak with a retention time of ~ 9 min [file 12967_2017_1351_MOESM1_ESM.tif]
